# Apparent coordinated and communal hunting behaviours by Erabu sea krait *Laticauda semifactiata*

**DOI:** 10.1038/s41598-023-48684-3

**Published:** 2023-12-06

**Authors:** Ruchira Somaweera, Vinay Udyawer, A. A. Thasun Amarasinghe, Joe de Fresnes, Jay Catherall, Galina Molchanova

**Affiliations:** 1 School of Biological Sciences, The University of Western Australia, Crawley, WA 6009 Australia; 2 Stantec Australia, Perth, WA 6000 Australia; 3 Australian Institute of Marine Science, Darwin, NT Australia; 4 Research Center for Biosystematics and Evolution, National Research and Innovation Agency (BRIN; Government of Indonesia), Cibinong, Indonesia; 5 Rivervale, Australia; 6 Wild Scuba Indonesia, Belongas Bay, Lombok, Indonesia

**Keywords:** Herpetology, Behavioural ecology

## Abstract

Opportunistic observations of Erabu sea kraits (*Laticauda semifaciata*) provide evidence that this species undertake a novel foraging tactic; coordinated communal hunting. Erabu sea kraits prey on cryptic fish species in highly complex reef habitats. Intra- and interspecific cooperative hunting strategies may increase chances for all members of the hunting party to encounter and capture prey in these complex habitats. Here, we observed 52 instances of communal hunting by Erabu sea kraits with conspecifics and other predatory fishes at recreational dive sites in Southern Lombok, Indonesia. These observations highlight the potential higher cognitive capacity of sea kraits to coordinate activities around communal hunting events.

## Introduction

Coordinated and communal hunting by a group of predators involves each individual within that group synchronising their actions in time and space to increase successful prey capture^1^. These behaviours have recently gained considerable attention as such coordination requires elevated cognitive demands. Role differentiated, coordinated and communal hunting is rare among animals, and are only known from a handful of mammals including primates, canines, hyenas, felines and cetaceans^2,3^, birds including raptors and corvids^4^ and fish including moray eels and trout^5,6^. Very limited instances of opportunistic, coordinated and communal hunting however has also been recorded in reptiles, including crocodilians^7^, varanids^8^, and a single case of a snake^9^. In the single observational study, Cuban boas (*Chilabothrus angulifer*) were suggested to take the positions of other individuals into account when choosing the hunting location for fruit bats, improving the effectiveness of the hunt. Here we describe possible coordinated communal hunting behaviour of a less-known marine snake, the Erabu sea krait.

Erabu or Chinese sea kraits (*Laticauda semifasciata*) are typically distributed in the tropical and subtropical waters around Japan, China including Taiwan, South Korea, Philippines and Indonesia^10^. It is possibly the most aquatic of all the amphibious sea kraits within the Laticaudid genera and live a nearly fully aquatic life except when laying eggs. Day time observations of this species during previous surveys have been in deep water (depth not specified but sampling undertaken on SCUBA), however, they seem to move to intertidal areas and coastal tidal caves at night time^11^, but always remain submerged^12^. This species relies on freshwater seepage and heavy rain for freshwater supply^13^ and availability of freshwater sources may be a determination of their distribution^14^. Despite the highly mobile nature of other members of their genera and their capacity to regularly move between neighbouring islands^15,16^, Erabu sea kraits shows distinctive genetic structure between island groups within their range^17^.

Specimen dissection studies at a local scale have shed some insight to the diet of Erabu sea kraits. At Orchid Island in Taiwan, stomach contents from 73 specimens recorded 16 fish families^11^. Hatchlings only ate fish of Mugiloididae, while subadult and mature kraits fed mainly on the Emmelichthyidae, Acanthuridae, and Pomacentridae, with mature males showing a wider range of food items (15 families) than adult females (6 families). Additionally, Bacolod^18^ briefly described females always having eels and other types of fish in their stomachs while Pickwell^19^ observed them reacting to smell of killifish (*Fundulus parvipinnis*) and mud suckers (*Gillichthys mirabilis*) under captive conditions. The cryptobenthic and reef-associated nature of the majority of preferred prey of Erabu sea kraits means that foraging and prey capture success is likely influenced by the complexity of habitat^20^, with lower capture success in highly structured and complex reef systems. In these instances, inter- and intra-specific coordinated group hunting likely increase the chances of prey capture^21^. Here we use opportunistic field observations, anecdotal records, and historical video records to describe apparent cooperative hunting by Erabu sea kraits, potentially used to increase prey capture success.

## Methods

Observations of foraging Erabu sea kraits were made during 32 recreational dives at ‘the Cathedral’ (− 8.899757°, 116.073019°) in Belongas, south of Lombok Island in Indonesia. The site, located 650 m off the closest shore, comprises a tall pinnacle surrounded by boulders reaching ~ 55 m in depth. It experiences high waves and strong currents with a surface water temperature of 27–30 and 25–27 °C at the bottom throughout the year.

Deep-diving certified scuba divers in groups of two to six visited the site 32 times between September 2018 and July 2022 during recreational dive tours. Once encountered, individuals of Erabu sea kraits were opportunistically observed from a distance of ~ 3 m (unless the kraits approach the divers) and followed for 5–20 min durations based on the dive conditions and behaviour of the kraits (e.g., when kraits swam away from the reef, or into depths divers were unable to follow). On some occasions, encounters were filmed with GoPro cameras without external lights. This work purely comprises a collation of opportunistic observations without interference made during recreational diving tours, therefore no animal ethics approval was obtained specifically for the work. However, standard guidelines for responsible animal interactions while scuba diving was strictly followed. As kraits were encountered during pre-planned recreational deep dives (> 20 m), depth and time restrictions meant that observers could not follow the groups of kraits through the full sequence of hunting behaviours in one dive. Nevertheless, repeated behaviours were noted across all observations and used to develop an ethogram highlighting key behaviours exhibited during apparent communal hunting events.

## Observations

Erabu sea kraits were encountered on at least 52 separate occasions, during all 32 dives. Of the 52 observations 12 were filmed, with the majority being anecdotal records (observed by JC and GM during recreational dives). The number of individuals per dive ranged from two individuals on 21 June 2022 to 21 individuals on 5 October 2019, and included individuals ~ 100 to ~ 130 cm in estimated total length. All encounters were between 23 and 45 m in depth. Common behaviours recorded during the encounters included at least 11 unique behaviours (Table 1). Due to the anecdotal nature of most observations, detailed time-budgets for the full sequence of behaviours could not be collected, however descriptions of components of interspecific and intraspecific communal hunting are provided here. Forty-three separate encounters were of swimming or foraging individuals and nine instances were of resting individuals (where individuals were drifting motionless along the substrate before being approached by divers; Table 1), five instances on a sandy bottom at ~ 42 m depth, and the rest among the reef structures ~ 25 m depth. Instances of both intraspecific and interspecific communal hunting was observed with some level of apparent coordination. Additionally, the sympatric yellow-lipped sea krait (*L. colubrina*) was encountered on three dives, but all individuals of this species were observed to be solitary and at depths of < 20 m.

**Table 1:** Ethogram of behavious observed during the course of 52 encounters of Erabu sea kraits (*Laticauda semifaciata*) undertaking apparent communal hunting during recreational dives at ‘the Cathedral’ dive site in southern Lombok, Indonesia between September 2018 and July 2022.

Behavioural observation	Description	Drawing
Travelling	Swimming > 2 m above substrate	
	Head pointed forwards	
	Directed movements	
Search swimming	Swimming < 2 m above reef structure	
	Head tilted down towards the reef	
	Tongue flicking	
Following (inter-specific)	Swimming closely behind another sea krait that is conducting prey searching behaviour	
	Swimming < 2 m above reef structure	
	Head pointed towards lead krait	
	Tongue flicking	
Following (intra-specific)	Swimming closely behind/among other predatory fish (usually larger than the krait)	
	Swimming < 2 m above reef structure	
	Head pointed towards predatory fish or towards direction of crevice with prey	
	Tongue flicking	
Investigating crevices	Sitting on substrate or parts of body touching the substrate	
	Head moving in and out of crevices	
	Tongue flicking	
Prey capture (within reefs)	Head within reef crevice	
	Sharp movement into the reef to capture prey	
	Tail flicking to maneuver	
	Exiting reef crevice with prey in mouth or in process of swallowing	
Prey capture (outside reef)	Quick swimming towards prey fish	
	Tongue flicking and head pointing towards prey	
	Striking at prey, often holding in position till prey succumbs to venom	
	Handling the prey to maneuver it into position for swallowing	
	Swallowing prey	
Postprandial behaviour	Reduces activity and moves back to travelling behaviour	
	Often descends to substrate with full body on substrate	
	Prey can often be observed moving down esophagus	
	In some instances individuals were observed re-adjusting their jaws by displaying a ‘yawn’ behaviour	
Resting	Full body on substrate and motionless	
	Often head within reef structure	
Other	Other non-predatory behaviour not catagorised above (e.g., copulation)	
	Surfacing for air	
	Amphibious behaviour, movements on land	
Not visible	Behaviours that are not visible to observer (i.e., within reef crevices)	

### Intraspecific coordinated and communal hunting

**Figure 1:**
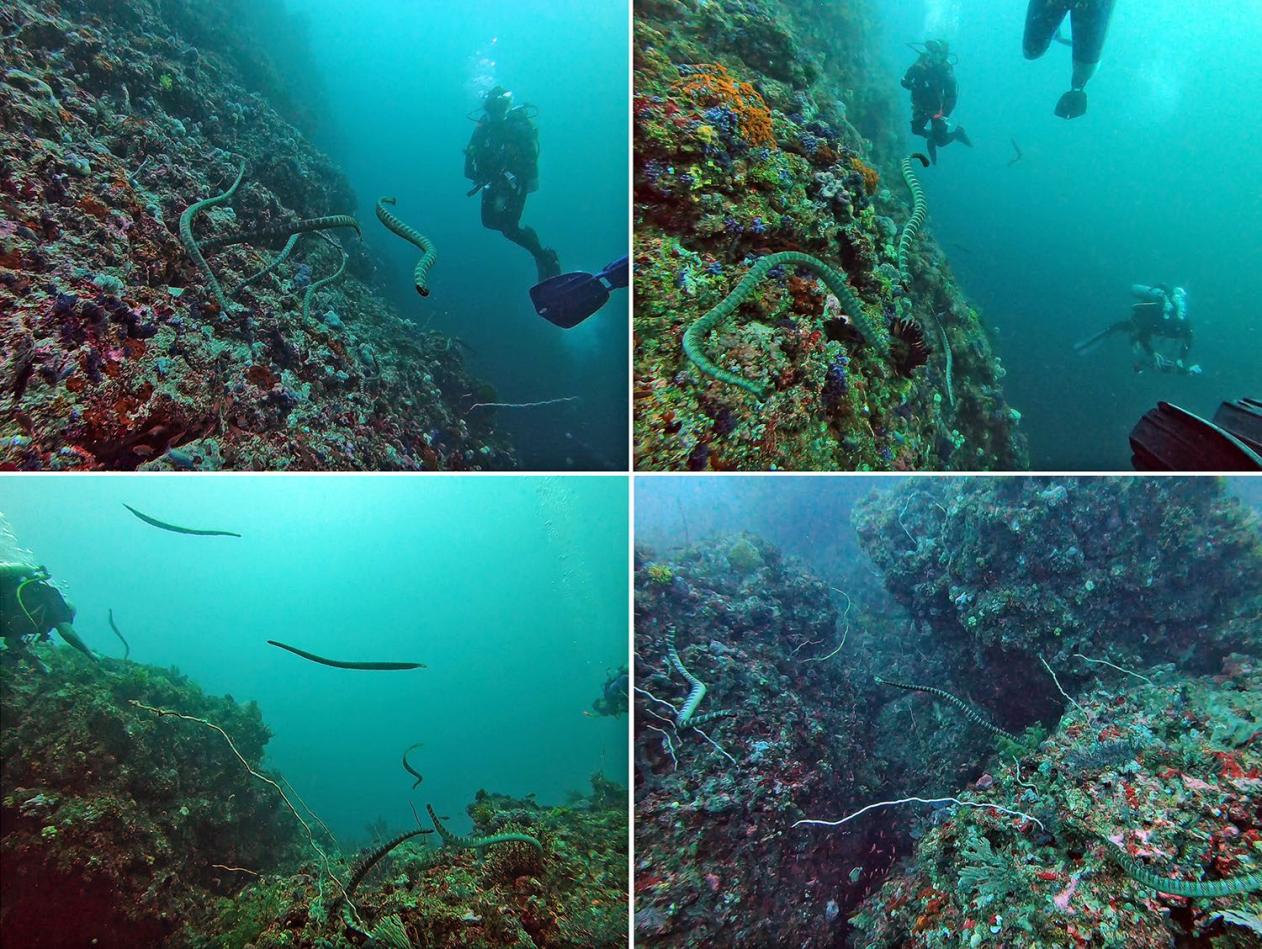
Records of intraspecific cooperative hunting by groups of Erabu sea kraits (*Laticauda semifaciata*) during recreational dives at ‘the Cathedral’ dive site in Southern Lombok, Indonesia. Sea kraits were observed swimming slowly in groups along a section of reef displaying cooperative hunting by focal kraits flushing prey out from cervices while conspecifics following behind.

On multiple occasions, two to six kraits were moving in closely bound groups (< 2 m apart) parallel to each other and to the reef at a ~ 0.5 to 2 m distance from the reef (Fig. 1). Individuals largely seemed to follow each other with the members of the front of the group located more closer to the reef than those in the back. Once those in the front of the group entered crevices and holes in the reef, those in the back remained outside at 1–2 m distance from the crevices rather than continuing swimming or exploring other crevices. Our observations were highly opportunistic, but at least in one instance, while two individuals exited the crevices, three others started searching new crevices, while those that did the prior searches followed the new search group. Entering of kraits into the crevices visibly flushed the fish hiding in the crevices, but we did not clearly observe any instances of conspecifics that remained outside successfully capturing the escaping fish. Although a successful capture of prey was not observed in this occasion, likely due to the short observation time, the consistent following and stopping of the group as the lead krait interrogated crevices suggests there may be a benefit to individuals that hunt in a group.

### Interspecific coordinated and communal hunting

**Figure 2:**
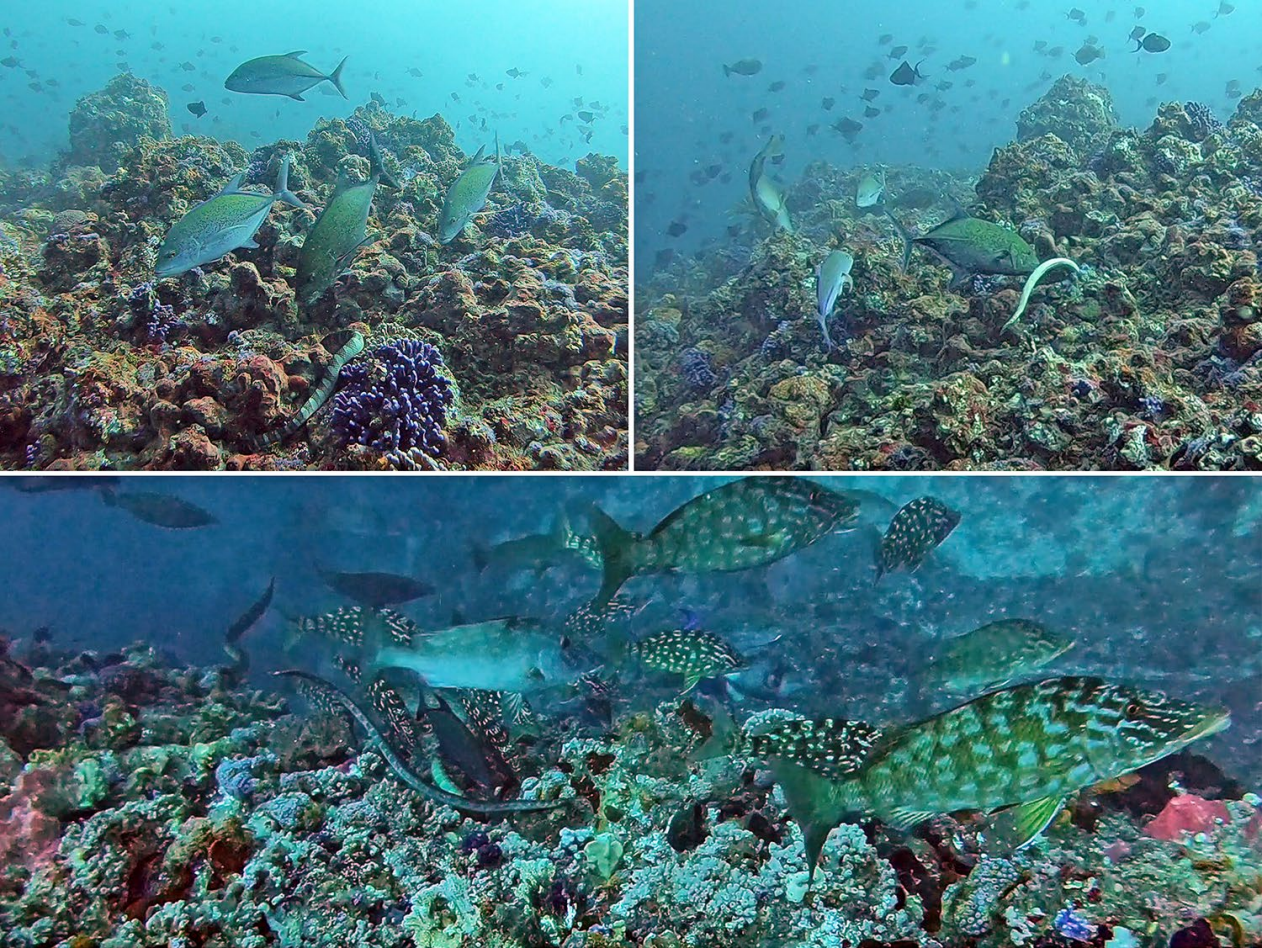
Records of interspecific group foraging between Erabu sea kraits and other predatory fish at dive sites in Southern Lombok. Predatory fish species that were observed to communally hunt with sea kraits included bluefin trevally (*Caranx melampygus*; top) and longface emperors (*Lethrinus olivaceus*; bottom).

On seven occasions, up to four kraits were observed foraging with bluefin trevally (*Caranx melampygus*) and in three occasions with longface emperors (*Lethrinus olivaceus*) (Fig. 2). These observations were relatively rare, however there was a clear distinction in roles played by the sea kraits and fish during these foraging events. In most cases, sea kraits foraged within cervices, while fish coordinated movements closely to follow the kraits and capture prey fish that escaped from the reef crevices. In one instance, a krait undertaking search swimming behaviour abruptly stopped over a coral crevice where three bluefin trevally were searching for prey. The krait commenced investigating the crevice and seemed to successfully capture prey in the reef crevice, which changed the behaviour of the trevally to hover directly over the krait as it commenced feeding. Each trevally seemed to compete for the position close to the opening of the crevice the krait was investigating, presumably to capture any other escaping fish, with one trevally driving the other individuals away. In these cases, it was difficult to determine if the predatory fish played any active role in the communal hunts (in contrast to just following kraits), however their presence along the reef pushed the prey fish into the reef and presumably increased the prey capture success for kraits within the reef crevices.

## Discussion

Known strategies of foraging in sea snakes range from ‘float and wait’ tactics, where pelagic snakes ambush small fish that congregate under floatsam^22^, to browsing across large areas to locate prey and food sources^23^, with foraging primarily considered solitary behaviours^24^. Foraging studies of sea snakes in the past have focused on what cues individual snakes utilise^25^, and how they use favorable environmental conditions to maximize prey detection and capture e.g., tidal cycles^26,27^. However, little has been recorded on the use of coordinated hunting tactics between conspecifics, and between other reef predators, to increase foraging success. In most instances, observations of sea snakes in other reef systems seem to suggest individuals rarely respond or react to conspecifics when in close proximity during foraging^24^, nevertheless synchrony in capture data of turtle-headed sea snakes (*Emydocephalus annulatus*) in New Caledonia suggest that, at least in that population, some form of cryptic social organisation does exists^28^. Weather this social organisation provides some advantages to foraging success of individuals, however, is still unknown.

There are several instances of snakes gathering for feeding due to a common stimulus. For example, dog-faced water snakes (*Cerberus rynchops*) and black-ringed mangrove sea snakes (*Hydrelaps darwiniensis*) gather in mangrove creeks in notable numbers during advancing and receding tides to feed on fish moving in narrow creeks (Somaweera pers. obs.), while multiple species of boids and colubrids form aggregations in cave passages to hunt bats entering and exiting roosts^29^. Galapagos racers (*Philodryas biserialis*) gather to hunt iguana hatchlings during hatching season (see https://youtu.be/B3OjfK0t1XM), while Australian scrub pythons (*Simalia amethistina*) seasonally congregate below communal rookeries of birds during nesting season^30^. However, field observations in these cases did not establish that the snakes take each other’s positions and/or actions into account during foraging. Therefore, it is difficult to confirm any form of coordinated feeding in these cases. Coordination is often assumed based on perceived complexity of hunting patterns. In all reported cases of apparent coordinated hunting in reptiles, some of the hunters drive the prey towards others, distract prey to facilitate the attack by others, or force prey into a compact area and then take turns attacking it^9^.

Coordinated or cooperative hunting requires the focal hunter to take the other hunters’ actions into account and therefore arguably requires complex cognitive ability^31^. Most of these hunts however are likely opportunistic where each individual attempts to increase the probability of catching the prey for itself. The limited, opportunistic observations made on Erabu sea kraits herein however suggests that potentially higher level of coordination is involved than simultaneous group hunting, given that individuals likely play different roles during a hunt. They are visually aware of the actions of the others in the group (e.g., remaining outside crevices when one group is hunting) and reacting accordingly (e.g., taking turns entering crevices). For some species and systems, It has been shown that coordinated hunts increase the effectiveness of prey capture, and thus increase food intake per individual^32^, but others have shown that this is not necessarily true^33^. It is possible that the communal behaviors such as these have other social functions.

Interspecific coordinated hunts between snakes and other reef predators are extremely rare. Anecdotal footage of sea snakes undertaking similar interspecific group hunting have been recorded elsewhere, e.g., group hunting by Erabu sea kraits with yellow goatfish (*Parupeneus cyclostomus*) and bluefin trevally (*Caranx melampygus*) in other locations in the Banda sea (see https://www.bbc.co.uk/programmes/p0038t09); between the closely related Katuali sea kraits (*Laticauda schistorhyncha*) and bluefin trevally in Niue (https://youtu.be/Q7pkazXvUG4); and between olive sea snakes (*Aipysurus laevis*) and coral trout (*Plectropomus leopardus*) on Ningaloo Reef, Western Australia (Hans Kemps, pers. obs.). In these cases, the kraits possibly play a similar function as moray eels (*Gymnothorax javanicus*) do in their recorded interspecies coordinated hunting observations with coral groupers (*Plectropomus pessuliferus*)^5^, where groupers corner prey into the reefs while eels flush cryptic prey out. The coordination between the two species results in mutually beneficial outcomes of increased prey capture success for both eels and groupers during the hunt.

Although opportunistic, our observations suggest that Erabu sea kraits potentially undertake intra- and inter-specific coordinated communal hunting. These observations indicate that by hunting in groups (with conspecifics or other predatory fishes), all members of the hunting party are likely to increase their chances of prey capture, however this is yet to be quantified. Here we have developed an ethogram of key behaviours observed, however there is a need to gain further information to build time-budgets for each of these behaviours and assess any observable patterns in communal and cooperative hunting in these species. Future research should also work to quantify the potential advantage Erabu sea kraits obtain from communal hunting. This could include the identification and longer-term tracking of individuals within hunting parties via marking and/or telemetry and quantifying if hunting in groups results in higher prey capture rates than solitary hunts. It also remains unclear whether involvement in these coordinated and communal hunts is an individual personality trait, and how individuals coordinate and synchronize their hunting. Our observations add to a growing body of literature on higher cognition levels than previously assumed among reptiles, specifically snakes.

## Data Availability

All data generated or analysed during this study are included in this published article.
